# Depression and heart failure associated with clinical COPD questionnaire outcome in primary care COPD patients: a cross-sectional study

**DOI:** 10.1038/npjpcrm.2014.66

**Published:** 2014-09-18

**Authors:** Manon Urff, Jan-Willem K van den Berg, Steven M Uil, Niels H Chavannes, Roger AMJ Damoiseaux

**Affiliations:** 1 Julius Centre for Health Sciences and Primary Care, University Medical Centre Utrecht, Utrecht, The Netherlands; 2 Department of Respiratory Medicine, Isala klinieken, Zwolle, The Netherlands; 3 Department of Public Health and Primary Care, LUMC, Leiden, The Netherlands; 4 General practice De Hof van Blom, Hattem, The Netherlands

## Abstract

**Background:**

Improvement in health-related quality of life (HRQoL) is one of the main goals in treating chronic obstructive pulmonary disease (COPD). Impaired HRQoL in COPD is associated with increased morbidity and mortality, hospitalisations and burden on our health-care system. The Clinical COPD Questionnaire (CCQ) is a validated, reliable, short questionnaire for the evaluation of disease-specific HRQoL in patients with COPD in primary care.

**Aims:**

To investigate factors that might be associated with CCQ outcome in COPD in a primary care setting.

**Methods:**

In a population of COPD patients in primary care, multiple regression analyses were used to assess associations between CCQ outcome and depression, heart failure, FEV_1_% predicted, FEV_1_/FVC, age, sex, body mass index and current smoking.

**Results:**

Data from 341 patients (mean age 68.1±10.3, COPD GOLD class I–III) were used for analyses. Together, heart failure and depression explained 23% of the variance in CCQ total score (*P*<0.001, *N*=93). Heart failure was most strongly associated with CCQ functional score (27% explained variance, *P*<0.001, *N*=100), whereas depression was most strongly associated with CCQ mental score (22% explained variance, *P*<0.001, *N*=93).

**Conclusions:**

CCQ outcomes are higher in COPD patients with heart failure and depression. These findings might imply that heart failure and depression affect HRQoL of patients with COPD, and thus emphasise the importance of a holistic approach of this complex disease, leading to a correct diagnosis of COPD and its comorbidities, to achieve better tailored treatment of chronic patients.

## Introduction

Chronic obstructive pulmonary disease (COPD) is a major cause of morbidity and mortality worldwide, and is estimated to become the third most common cause of death in 2020.^1,2^ Since health-related quality of life (HRQoL) is impaired in COPD, one of the main goals in COPD treatment is improving it.^2–7^ Impaired HRQoL has been shown to be associated with increased morbidity, mortality,^8^ hospitalisations^9^ and health-care consumption.^10–15^ These associations are independent of the level of airway obstruction measured by spirometry.^11,14^ Although spirometry has been regarded as the standard method for grading COPD severity, patient-reported outcomes should be used to provide information about the effect of COPD on patients’ daily life.^2,16^ Also, COPD treatment effects can be measured by disease-specific HRQoL questionnaires.^7,16^

The Clinical COPD Questionnaire (CCQ) is a validated, reliable, short questionnaire for the evaluation of disease specific HRQoL in patients with COPD.^17^ Several studies showed a weak association between CCQ and FEV_1_% predicted.^17–20^ The association between comorbidity and CCQ outcome remains to be assessed. If comorbidity would affect CCQ outcome, treatment effects specific for COPD might be masked. On the other hand, COPD outcome could be improved by specifically tackling comorbidity.

In general there is some evidence that comorbidity in COPD patients is associated with worse HRQoL scores in HRQoL measurements specific for COPD.^12,21–23^ Common comorbities in COPD are depression and heart failure, besides asthma, diabetes and osteoporosis. In this study we restricted our investigation to heart failure and depression because of clear overlapping symptoms assessed by the CCQ. Diagnosing heart failure in COPD may be difficult, as dyspnoea symptoms might unjustly be attributed to COPD in the presence of heart failure. Because signs and symptoms related to COPD also mimic the clinical presentation of heart failure, COPD-specific HRQoL measurements like the CCQ might be influenced by this comorbidity. As both diseases share matching risk factors, particularly cigarette smoking, simultaneous appearance is not unlikely. Prevalence of heart failure in the general European population is estimated between 0.4–2%.^24^ However, Rutten *et al.* ^25^ found a prevalence of heart failure in COPD of 26% in patients over 70 years of age, of which only 5.5% was actually recognised as heart failure. Prevalence of depression in the general population is estimated at 5.8%.^26^ In COPD patients, estimated prevalence ranges from 6–42%.^27,28^

This cross-sectional study investigated factors that might be associated with HRQoL measured by CCQ in a population of COPD patients in a primary care setting. Those factors were depression, heart failure, FEV_1_% predicted, FEV_1_/FVC, age, sex, body mass index (BMI) and current smoking.

## Materials and Methods

### Design and ethical consideration

We used a cross-sectional study design, using data records from daily general practice in the region of Zwolle (Netherlands) between 2009 and 2011. Seventeen general practitioners from urban and rural general practices participated in the ‘Extended care project for COPD’, a project for optimising regular COPD care.^29,30^ Data used for this study were anonymously collected from a database from the general medical practices that participated in this project. General practitioners gave permission for the use of these anonymously generated data for research purposes. Researchers were unable to connect the data to actual patients. Therefore, no informed consent or ethical approval was needed according to the Dutch Central Committee on research involving Human Subjects.^31^

### Population

For this study, we selected patients with COPD from the ‘Extended care project for COPD’ database. To identify all patients with (suspected) COPD in the participating general practices for the ‘Extended care project for COPD’, a systematic search in the electronic database of the participating general practitioners by using the Internal Classification of Primary Care code R95 COPD (ICPC-code R95) was performed.

In addition, patients were identified by searching for the abbreviation LO, which was used in the database for all lung diseases. Patients were also identified when they had two or more prescriptions of inhaled pulmonary medication in the previous year according to the Anatomic Therapeutic Chemical Classification list of prescribed medication in general practice. Patients with confirmed COPD who were primarily treated by a pulmonologist, were not included in the database.

All identified patients were invited to visit their general practice in connection with the ‘Extended care project for COPD’ to optimise their treatment. During this visit spirometry was obtained in all patients to confirm the diagnosis of COPD.

For this study, we selected only the patients with COPD from the ‘Extended care project for COPD’ database, on the basis of an FEV_1_/FVC <0.7 after bronchodilatation according to international guidelines.^2^

For the project, both the visit and the data collection were done by practice nurses, who were trained in spirometry testing according to current guidelines.^32^ All data per patient were preferably collected on the same day. In one practice, spirometry was performed in the lung function laboratory, without changing treatment in the period between spirometry and other measures, which was maximal 3 weeks (*N*=27, 7.9% of total population).

### Measurements

During the visit several variables were measured, of which the measurement method is described in the following paragraphs. The following variables were measured: CCQ total and domain scores, depression (yes/no), heart failure (yes/no), FEV_1_% predicted and FEV_1_/FVC (post-bronchodilator forced expiratory volume in 1 s/forced vital capacity, measured by spirometry), age (⩽65, 65–75, ⩾75), sex (male/female), BMI (kg/m^2^) and current smoking (yes/no) and COPD GOLD-classification (I–IV).

Spirometry was performed according to procedures and guidelines of the European Respiratory Society/American Thoracic Society (ERS/ATS), which is also used in the Netherlands.^32^ The COPD GOLD class was determined by spirometry according to the Global Initiative for Chronic Obstructive Lung Disease.^2^ GOLD I: FEV_1_⩾80% predicted, GOLD II: 50%⩽FEV_1_<80% predicted, GOLD III: 30%⩽FEV_1_<50% predicted, GOLD IV: FEV_1_<30% predicted.

The CCQ was completed by patients before the visit and was handed over to the practice nurse. The CCQ consists of three domains: symptoms (four items), mental (two items), and functional (four items). Each question is graded according to a 7-point Likert scale from 0 to 6.^17^ The total score is the sum of all item scores divided by 10. Lower scores indicate better HRQoL: a score of 0 indicating best possible HRQoL and 6 indicating worst HRQoL. The CCQ is externally validated and reliably tested in general practice populations in several countries, including the Netherlands.^17^ The minimal clinically important difference (MCID) was defined at 0.4 points.^33^ As the MCIDs of separate domains were never assessed (personal correspondence with Dr JWH Kocks), the MCID for the CCQ total score was used when evaluating the domain scores. The MCID was only used for comparison with the differences between subgroups to gain information about the clinical relevance of our findings.

Data regarding depression were collected from the practices’ electronic database using ICPC-code P76 for depression, which includes all patients with the clinical diagnosis of depression.

Heart failure was determined in the same way, by using ICPD-code K77 for heart failure; which includes all patients diagnosed with heart failure before this project was initiated. Patients with unknown information about heart failure and elevated pro-BNP in laboratory at the inclusion of this project, underwent transthoracic echocardiography in the workup for the project to confirm or reject the diagnosis of heart failure.

BMI definitions were based on the Dutch College of General Practitioners’s guidelines: underweight <18.5, normal weight=18.5–25, overweight=25–30 and obesity ⩾30).

### Statistical analyses

Descriptive statistics were used to characterise the population. Correlations between CCQ outcome and the variables depression, heart failure, FEV_1_% predicted, FEV_1_/FVC, age, sex, BMI and current smoking were assessed by calculating Pearson’s correlation coefficients (*R*_P_) in parametric variables and Spearman’s rank correlations coefficients (*R*_S_) in nonparametric variables. Furthermore, stepwise multiple regression analyses were conducted to assess relationships between CCQ outcome (total and domain scores) and variables which have shown a correlation (*P* value <0.15) with CCQ outcome.

Next, subgroups regarding depression were established to assess the magnitude of the differences in CCQ scores between the group of patients with depression and the group of patients without depression. This was also done for heart failure.

Student’s *t*-tests for independent groups were conducted to compare two subgroups, the nonparametric alternative was Mann–Whitney *U*-test. All analyses were performed with Statistical Package for the Social Sciences (SPSS) version 16.0 for Windows (SSPS, Chicago, IL, USA).

## Results

### Characteristics

**Figure 1:**
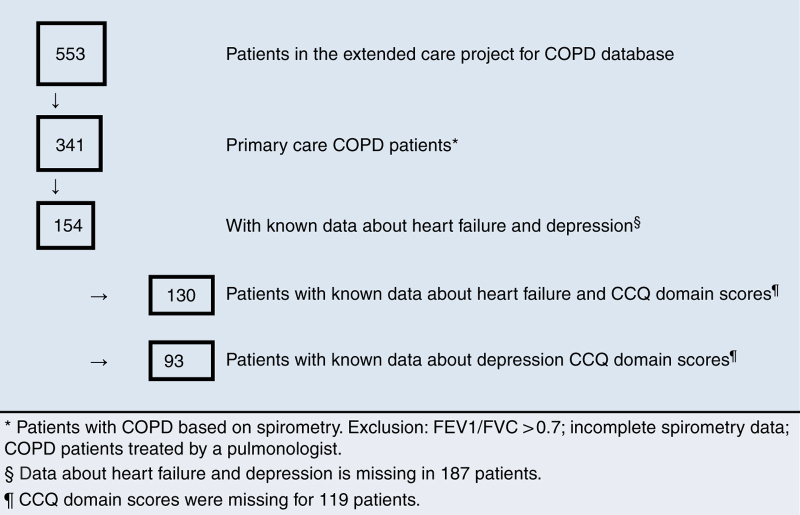
Flow chart.

From a total of 553 patients in the ‘extended care project for COPD’ database, who were selected as described in the Materials and Methods section, 341 patients were diagnosed with COPD on the basis of respiratory symptoms and spirometry (Figure 1). Characteristics of this group are shown in Table 1. Information about heart failure and depression was known for 154 patients. For the project it was intended that a pro-BNP would be measured in all patients with unknown information about heart failure: unfortunately this is not routinely done. Next to this, in 119 patients, data about CCQ domain scores were missing.

Because of this missing data, parts of the analysis were done in fewer patients (93, 100, 130, respectively for CCQ mental score, CCQ functional score, CCQ total score). We compared basic characteristics from the total study group (*N*=341) with the groups with data about depression (*N*=154), heart failure (*N*=154) and CCQ domain scores (*N*=222). We found a difference in smoking status (37% smokers in the group with data about heart failure, 40.9% smokers in the group with data about depression, compared with 30.2% smokers in the total study group), other parameters showed no differences.

### Correlations

Our results showed significant correlations between CCQ total score and FEV_1_% predicted (*R*_P_=−0.13, *P*=0.015), heart failure (*R*_S_=0.31, *P*<0.0001) and depression (*R*_S_=0.21, *P*=0.010).

### Multiple regression analysis

For multiple regression analysis with CCQ outcome as the dependent variable, the other variables were selected on the basis of a correlation with *P*<0.15. For the CCQ total score as the dependent variable, FEV_1_% predicted, sex, age, depression and heart failure were selected. Similarly, for CCQ symptom score, FEV1% and depression were selected; for CCQ mental score, depression, FEV_1_/FVC, FEV_1_% predicted, age and sex were selected; for CCQ functional score, heart failure, FEV_1_% predicted and BMI were selected.

There was a significant association between the CCQ total score and depression and heart failure (*P*<0.0001), in which heart failure and depression explained 23% of the CCQ total score variance. Heart failure alone explained 19% of the CCQ total score variance (Table 2). FEV_1_% predicted, gender and age were not significantly associated with the CCQ total score in this model (Table 2).

The CCQ symptom score was also significantly associated with depression, which explained 4.7% of the CCQ symptom score variance (*P*<0.0001, not shown). FEV_1_% predicted was not significantly associated with the CCQ symptom score in this model.

Depression and FEV_1_/FVC explained 26% of the CCQ mental score variance (*P*<0.0001, Table 3). FEV_1_% predicted, age and sex were not significantly associated with the CCQ mental score.

Heart failure explained 27% of variance of the CCQ functional score (*P*<0.0001, Table 4). FEV_1_% predicted and BMI were not significantly associated with the CCQ functional score in this model.

### Subgroup analyses

COPD patients with heart failure had significantly higher CCQ scores than COPD patients without heart failure (Table 5). The largest mean difference between both groups was found in CCQ total scores (1.92 vs. 1.23; mean difference=−0.70; 95% confidence interval (CI)=−1.00, −0.40; *P*<0.0001) and in CCQ functional scores (2.33 vs. 1.22; difference=−1.31; 95% CI=−1.92, −0.69; *P*<0.0001).

COPD patients with depression also had higher CCQ scores than COPD patients without depression (Table 6). The largest mean difference between both groups was found in CCQ total scores (1.92 vs. 1.30; difference=−0.63; 95% CI=−0.99, −0.27; *P*=0.018) and in CCQ mental scores (1.53 vs. 0.42; difference=−1.11; 95% CI=−1.82, −0.39; *P*=0.005). In CCQ symptom scores, the mean difference was −0.61 (2.59 vs. 1.98, 95% CI=−1.18, −0.03; *P*=0.038).

## Discussion

### Main findings

We found that heart failure and depression were associated with CCQ scores. To be precise, 27% of the variance in CCQ functional score was explained by heart failure and 22% of the variance in CCQ mental score was explained by depression. Subgroup analysis, although unadjusted, showed that COPD patients with depression or heart failure had considerably higher CCQ scores, indicating worse HRQoL, than COPD patients without these co-morbidities.

### Interpretation of finding in relation to previously published work

Recent literature showed weak associations between CCQ score and FEV_1_% predicted in primary care.^17–19,23^ However, few studies performed analysis with adjustment for co-variables by multiple regression analysis.^34^ Van der Molen *et al.* ^17^ and Damato *et al.* ^18^ found correlations between CCQ total score and FEV_1_% predicted of −0.49 and −0.57, respectively. Their study populations consisted of healthy people as well as COPD patients and they only applied correlations, without adjusting for co-variables by multiple regression analysis.^17,18^ Reda *et al.* ^19^ found correlations between CCQ total, symptom, mental and functional score and FEV_1_% predicted in smoking COPD patients in primary care of −0.19, −0.20, −0.18, −0.12, respectively (all not significant), compared with −0.13, 0.02, −0.10 and −0.12 in our population (only CCQ total score significant). In our population a noteworthy proportion of COPD patients quit smoking after diagnosis; at the time of study they were nonsmokers (with a history of smoking). Reda *et al.* ^19^ possibly missed a part of COPD patients in their analysis, because they only included COPD patients who were smokers at the time of inclusion and excluded COPD patients who were nonsmokers at the time of inclusion. As a result, their results may not be representative for all COPD patients (smokers, nonsmokers and patients who quit smoking after diagnosis) in primary care.

We found that heart failure and depression were associated with CCQ outcome. These results are in line with earlier findings, Sundh *et al.* ^23^ also performed adjustment for lung function in a subgroup, but lung function tests were not performed at the same time (interval several years) as the CCQ and can therefore bias the results. The mean CCQ in the study of Sundh *et al.* ^23^ was higher than in our study, indicating a worse COPD population. The distribution of the different GOLD categories in primary care in the study of Sundh is about the same as the distribution of all patients with COPD in the Netherlands.^35^ As we excluded patients treated by a pulmonologist, it is obvious that COPD in our population was less severe.

The association we found might imply that patients with COPD combined with depression or heart failure have worse COPD or that due to depression and heart failure HRQoL (measured by CCQ) is worse.

Heart failure was most strongly associated with CCQ total and functional score, which was quantified by significantly worse CCQ total and functional scores in the group COPD patients with heart failure, compared with those without heart failure. The difference between groups was 0.70 and 1.31, respectively, which exceeded the MCID of the CCQ total score. The higher CCQ functional score in patients with heart failure can possibly be explained by corresponding symptoms of COPD and heart failure, as reflected in the questions of the CCQ. Patients with both diseases probably cannot distinguish between symptoms caused by COPD and heart failure, so the CCQ score is likely affected by the patients’ symptoms of heart failure. This shows that knowledge about comorbidities is important for the interpretation of the CCQ (and possibly also other HRQoL measurements), but also offers new treatment opportunities. If comorbidities are managed better, HRQoL outcomes in COPD might potentially improve.

The association between depression and CCQ outcome was seen in CCQ total, symptom and mental score and was quantified by subgroup analysis, which showed significantly higher CCQ scores in COPD patients with depression compared with COPD patients without depression. The difference exceeded the MCID of CCQ total score in all domains that were associated with depression. The higher CCQ mental score in COPD patients with depression can be explained by symptoms of depression that correspond to questions on the mental domain of CCQ. Furthermore, a possible explanation for the association between depression and CCQ symptom score is that patients with depression perceive their symptoms worse, or they actually have more symptoms.

### Strengths and limitations of this study

Our study population is representative of the population of COPD patients who are treated in primary care in the Netherlands. Unfortunately, a consequence of using data from daily practice is that in some patients with the diagnosis COPD, depression and heart failure, data were missing. A number of statistical analyses were therefore done with data from fewer (93–130 patients) than the total of 341 patients. This could have introduced some bias if the missings were systematic. However, it was impossible to detect whether in the case of missing comorbidity data, the presence of heart failure and depression were not known or that data were not recorded for the project. We found that the patient groups with data about heart failure and depression had few more smokers compared with the total study group. However, in the total study group, there were more patients with missing data on smoking status, so it remains unclear if there actually were more smokers in the smaller groups.

Our study showed a prevalence of heart failure of 8.2%. Like in the study of Rutten *et al.*,^25^ our population probably also consisted of COPD patients with unrecognised heart failure, although the mean age in our population was lower. The patients with unrecognised heart failure in our study were never diagnosed with heart failure and therefore analysed in the group of patients without heart failure, so the influence of heart failure on the CCQ outcome in COPD might be underestimated.

Prevalence of depression in our study was 6.5%; this is the lower limit of prevalence shown in literature, so there is a possibility that a proportion of COPD patients with depression were in the group of COPD patients without depression.^27,28^ This might have led to underestimation of the strength of the association between depression and CCQ outcome in COPD, as well.

A part of patients’ data was incomplete as CCQ domain scores were missing. To be certain that there was no difference between the groups with or without domain scores, we compared CCQ total scores with a Student’s *t*-test and found no significant difference.

### Implications for future research, policy and practice

Because attention for COPD in primary care is increasing, knowledge is needed on how to improve COPD care and how to reach the COPD guidelines’ goals for treating COPD. First, improving HRQoL is one of the main goals in treating COPD.^2^ Factors associated with HRQoL outcome in COPD have been increasingly studied.^10,12,15,21–23,34,36^ We showed that CCQ outcome is higher in patients with COPD combined with heart failure and depression. The fact that the CCQ outcome is higher in patients with COPD combined with heart failure or depression, must make us alert when evaluating treatment effects in COPD. Knowledge of comorbidities in COPD is important for the correct interpretation of the CCQ outcomes. Also, treating these relevant comorbidities might improve HRQoL in COPD.

Further (prospective) research should be done to estimate the influence of depression, heart failure and other comorbidities on CCQ in order to confirm our results, in the search for improved tailored treatment of COPD.

### Conclusions

The CCQ is a disease-specific HRQoL questionnaire to evaluate clinical control of COPD in daily practice.^17^ We found that CCQ scores are higher in COPD patients with heart failure and depression. These findings imply that heart failure and depression affect HRQoL of patients with COPD, and thus emphasise the importance of a holistic approach of complex disease, leading to a correct diagnosis of COPD and its comorbidities to achieve better tailored treatment of chronic patients.

## Figures and Tables

**Table 1:** Patient characteristics

	*% Or mean (s.d.)*	N
*Sex*		
Male	61.6	210
Female	38.4	131
Age (in years)	68.1 (10.3)	341
Body mass index (kg/m^2^)	27.1 (4.7)	341
FEV_1_/FVC post-bd	0.56 (0.1)	341
FEV_1_ post-bd % predicted	71.5 (15.9)	341
		
*CCQ score*		
Total	1.3 (0.8)	341
Symptom	1.9 (1.1)	222
Mental	0.5 (0.7)	222
Functional	1.1 (0.9)	222
		
*GOLD-class (*N*=341)* a		
I; FEV_1_⩾80% predicted	29.9	102
II; 50%⩽FEV_1_<80% predicted	63.1	215
III; 30%⩽FEV_1_<50% predicted	7.0	24
IV; FEV_1_<30%	0	0
		
*Current smoking (*N=*298)*		
Yes	30.2	103
No	57.2	195
Missing	12.6	43
		
*Heart failure*		
Yes	8.2	28
No	37.0	126
Missing	54.8	187
		
*Depression*		
Yes	6.5	22
No	38.7	132
Missing	54.8	187

Abbreviations: CCQ, Clinical COPD Questionnaire; FEV_1_/FVC post-bd, post-bronchodilator forced expiratory volume in 1 s/forced vital capacity.

a GOLD-class I–IV explained in the Materials and methods section.

**Table 2:** Regression model for CCQ total score^a^

*Model*	*Independent variable*	R	R^*2*^	*ANOVA*		*B*	P *value*	*95% CI for B*	
				*F*	P *value*			*Lower*	*Upper*
1^b^		0.44	0.19	30.84	<0.0001				
	Constant					1.21	<0.0001	1.07	1.34
	Heart failure					1.06	<0.0001	0.68	1.43
2^c^		0.48	0.23	18.82	<0.0001				
	Constant					1.17	<0.0001	1.03	1.30
	Heart failure					1.00	<0.0001	0.63	1.37
	Depression					0.51	0.02	0.09	0.93

*R* is the measure of the correlation between the observed value and the predicted value of the dependent variable. *R* ^2^ indicates the proportion of the variance in the dependent variable which can be explained by the independent variables, Β indicates values for the regression equation for predicting the dependent variable from the independent variable.

Abbreviations: ANOVA, analysis of variance; CCQ, Clinical COPD Questionnaire; CI, confidence interval.

a Stepwise regression model with dependent variable: CCQ-total score; *N*=130 for model 1, *N*=93 for model 2.

b Predictor in model 1: heart failure. Excluded during analysis: depression, FEV_1_%predicted, gender, age.

c Predictors in model 2: heart failure, depression. Excluded during analysis: FEV_1_%predicted, gender, age.

*P*<0.05 is considered significant (two-tailed).

**Table 3:** Regression model for CCQ mental score^a^

*Model*	*Independent variable*	R	R^*2*^	*ANOVA*		*B*	P *value*	*95% CI for B*	
				*F*	P *value*			*Lower*	*Upper*
1^b^		0.46	0.22	24.88	<0.0001				
	Constant					0.42	<0.0001	0.24	0.61
	Depression					1.11	<0.0001	0.67	1.55
2^c^		0.51	0.26	16.04	<0.0001				
	Constant					1.46	0.001	0.59	2.33
	Depression					1.11	<0.0001	0.68	1.54
	FEV_1_/FVC					−0.19	0.017	−0.035	0.0034

*R* is the measure of the correlation between the observed value and the predicted value of the dependent variable. *R* ^2^ indicates the proportion of the variance in the dependent variable which can be explained by the independent variables, Β indicates values for the regression equation for predicting the dependent variable from the independent variable.

Abbreviations: ANOVA, analysis of variance; CCQ, Clinical COPD Questionnaire; CI, confidence interval; FEV_1_/FVC, forced expiratory volume in 1 s/forced vital capacity.

a Stepwise regression model with dependent variable CCQ-mental score; *N*=93.

b Predictor in model 1: depression. Excluded during analysis: FEV_1_/FVC, FEV_1_%predicted, age, gender.

c Predictors in model 2: depression, FEV_1_/FVC. Excluded during analysis: FEV_1_%predicted, age, gender.

*P*<0.05 is considered significant (two-tailed).

**Table 4:** Regression model for CCQ functional score^a^

*Model*	*Independent variable*	R	R^*2*^	*ANOVA*		*B*	P *value*	*95% CI for B*	
				*F*	P *value*			*Lower*	*Upper*
1^b^		0.52	0.27	34.89	<0.0001				
	Constant					1.00	<0.0001	0.81	1.20
	Heart failure					1.35	<0.0001	0.90	1.80

*R* is the measure of the correlation between the observed value and the predicted value of the dependent variable. *R* ^2^ indicates the proportion of the variance in the dependent variable which can be explained by the independent variables, Β indicates values for the regression equation for predicting the dependent variable from the independent variable.

Abbreviations: ANOVA, analysis of variance; CCQ, Clinical COPD Questionnaire; CI, confidence interval.

a Stepwise regression model with dependent variable: CCQ-functional score; *N*=100.

b Predictor in model 1: heart failure. Excluded during analysis: FEV1%, BMI.

*P*<0.05 is considered significant (two-tailed).

**Table 5:** Mean (s.d.) CCQ scores in primary care COPD patients with and without heart failure^a^

	*Heart failure (*N*=28)* b	*No heart failure (*N*=126)* c	*Mean difference*	*95% CI*		P *value*
				*Lower*	*Upper*	
CCQ total score	1.92 (0.9)	1.23 (0.7)	−0.70	−1.00	−0.40	<0.0001
CCQ symptom score	2.30 (1.1)	1.95 (1.0)	−0.34	−0.85	0.16	0.182
CCQ mental score	0.74 (1.0)	0.49 (0.7)	−0.25	−0.64	0.15	0.219
CCQ functional score	2.33 (1.2)	1.02 (0.8)	−1.31	−1.92	−0.69	<0.0001

Abbreviations: CCQ, Clinical COPD Questionnaire; CI, confidence interval; COPD, chronic obstructive pulmonary disease.

a Student’s *t*-test. *P*<0.05 is considered significant.

b *N*=19 in CCQ domain scores.

c *N*=81 in CCQ domain scores.

**Table 6:** Mean (s.d.) CCQ scores in primary care COPD patients with and without depression^a^

	*Depression (*N*=22)* b	*No depression (*N*=132)* c	*Mean differences*	*95% CI*		P *value*
				*Lower*	*Upper*	
CCQ total score	1.92 (1.1)	1.30 (0.7)	−0.63	−0.99	−0.27	0.018
CCQ symptom score	2.59 (1.3)	1.98 (1.0)	−0.61	−1.18	−0.03	0.038
CCQ mental score	1.53 (1.4)	0.42 (0.7)	−1.11	−1.82	−0.39	0.005
CCQ functional score	1.59 (1.3)	1.15 (0.9)	−0.44	−1.14	0.26	0.209

Abbreviations: CCQ, Clinical COPD Questionnaire; CI, confidence interval; COPD, chronic obstructive pulmonary disease.

a Student’s *t*-test. *P*<0.05 is considered significant.

b *N*=17 in CCQ domain scores.

c *N*=76 in CCQ domain scores.
